# An intra-K-complex oscillation with independent and labile frequency and topography in NREM sleep

**DOI:** 10.3389/fnhum.2013.00163

**Published:** 2013-04-26

**Authors:** Vasileios Kokkinos, Andreas M. Koupparis, George K. Kostopoulos

**Affiliations:** ^1^Neurophysiology Unit, Department of Physiology, Medical School, University of PatrasPatras, Greece; ^2^Epilepsy Monitoring Unit, St. Luke's HospitalThessaloniki, Greece

**Keywords:** K-complex, NREM sleep, human EEG, theta, alpha

## Abstract

NREM sleep is characterized by K-complexes (KCs), over the negative phase of which we identified brief activity in the theta range. We recorded high resolution EEG of whole-night sleep from seven healthy volunteers and visually identified 2nd and 3rd stage NREM spontaneous KCs. We identified three major categories: (1) KCs without intra-KC-activity (iKCa), (2) KCs with non-oscillatory iKCa, and (3) KCs with oscillatory iKCa. The latter group of KCs with intra-KC-oscillation (iKCo), was clustered according to the duration of the iKCo. iKCa was observed in most KCs (1150/1522, 75%). iKCos with 2, 3, and 4 waves were observed in 52% (786/1522) of KCs in respective rates of 49% (386/786), 44%, and 7%. Successive waves of iKCos showed on average a shift of their maximal amplitude in the anterio-posterior axis, while the average amplitude of the slow KC showed no spatial shift in time. The iKCo spatial shift was accompanied by transient increases in instantaneous frequency from the theta band toward the alpha band, followed by decreases to upper theta. The study shows that the KC is most often concurrently accompanied by an independent brief iKCo exhibiting topographical relocation of amplitude maxima with every consecutive peak and transient increases in frequency. The iKCo features are potentially reflecting arousing processes taking place during the KC.

## Introduction

The K-complex (KC) is a major EEG graphoelement characterizing the second stage of the human NREM sleep. By definition, the KC is a biphasic slow wave that stands out of the NREM electroencephalographic background, with a negative phase that may or may not be immediately followed by a positive phase or a sleep spindle (Colrain, 2005; Halász, 2005). The KC was initially described more than 70 years ago by Loomis et al. (1938), but its functional role is still not clearly determined. Yet several of its properties are well-documented: it can be emitted spontaneously as well as be evoked by external stimuli (Loomis et al., 1938; Niiyama et al., 1996), it is exclusive to NREM sleep (Weitzman and Kremen, 1965; Goff et al., 1966), it has a primarily frontal lobe distribution (Davies et al., 1939; Massimini et al., 2004), it is accompanied by autonomic alterations (Roth et al., 1956; Ackner and Pampiglione, 1958; Hornyak et al., 1991; Okada et al., 1991; Takehuchi et al., 1994; Tank et al., 2003), it is a phenomenon independent of sleep spindles (Johnson et al., 1976; Church et al., 1978), its frequency of occurrence is decreased by benzodiazepine administration (Gaillard and Tissot, 1975; Johnson et al., 1976, 1979; Naitoh et al., 1982; Kubicki et al., 1987) and its neuronal generators are cortical and widely, although predominantly frontally, distributed (Velasco et al., 2002; Cash et al., 2009).

The main question regarding the KC along the course of sleep research has been whether it represents a brief hypnagogic or arousing neuronal brain function. It is believed to introduce delta activity into NREM sleep (Halász et al., 1977; Halász, 1981; De Gennaro et al., 2000; De Gennaro and Ferrara, 2003) and for that it is considered to be a sleep-promoting mechanism. In particular, the spontaneous KC is considered to be reacting to unknown endogenous or exogenous stimuli that are not intense enough to provoke an arousal to full wakefulness (Halász et al., 1985). On the other hand, the KC is often followed by arousal- and microarousal-related EEG events (Ehrhart et al., 1981), autonomic alterations (Sforza et al., 2000), as well as movement (MacFarlane et al., 1996); therefore, it is considered by some to be a micro-arousing cortical reaction. As it is commonly agreed that the KC *per se* is unlikely to be responsible for the fluctuations observed in autonomic measurements, another argument has been put forward according to which the KC is a cortical reaction to stimuli that can also cause autonomic reactions, thereby aiming in avoiding cortical awakening, sometimes achieving sleep-protection and sometimes failing to (Colrain, 2005; Halász, 2005). Another theory considers the KC highly correlated to brief (<1 s) cortical depolarization-hyperpolarization oscillations, during which intra-cortical activity takes place as the cortex has been isolated from the environment by thalamic inhibition. Thereby, the KC could represent the transition between a state of neuronal activation (depolarization phase) and a state of rest (hyperpolarization phase) reflected in the KC prominent negative phase (Amzica and Steriade, 2002; Cash et al., 2009).

In a recent work investigating rhythmic activity around and during the KC (Kokkinos and Kostopoulos, 2011), we reported sleep spindle interruption upon coincidental KC appearance and generation of higher spectral frequency sleep spindles toward the falling negative/positive phase of the KC. In that report we also observed that during the time course of the KC negative phase, and independently of any coincidence/interruption of spindles, a brief oscillation at the upper limits of the theta band briefly appears around the negative peak of the KC. The present study focused in characterizing that intra-KC oscillatory (iKCo) activity as it hasn't yet been the object of systematic research. Describing events during the time-course of the KC may allow us to understand its physiological role, which has been proposed to relate to both the arousal level and to brain information processing (Colrain, 2005).

## Materials and methods

### Subjects and procedures

Seven individuals (4 females and 3 males) aged between 24 and 33 years (mean age 27.6 ± 3.31) participated in the sleep study. All volunteers were good sleepers, without difficulty in falling or remaining asleep during the night. All of them were in good health and free from medication at the time of study. None of the participants reported a history of neurological or psychiatric disorder, or disordered sleep. Subjects kept a 7-day sleep diary, they were instructed to follow their regular sleep schedule, as well as refrain from alcohol and caffeine at least 3 and 1 days, respectively, prior to the experiment. Menstrual phase was not controlled for in female subjects. All participants read and signed an informed consent form describing in detail the procedures and purposes of the sleep study.

Subjects arrived at the laboratory for electrode preparation approximately 1 h prior to their usual bedtime, the latter calculated as an average of bedtimes the last 7-days described in their sleep diaries. Each of them spent a whole night in the laboratory, in an air-conditioned soundproof temperature-controlled Faraday-cage dark room that was intentionally not video-monitored in order to avoid potential sleep disturbances. No pharmacological substance was administered in order to induce sleep. The sleep recording session begun after the subjects willingly switched off the room lights, as were instructed to do when they would feel like falling asleep, and ended with their spontaneous wake-up in the morning. All recorded electrophysiological signals were monitored in an adjacent room and the possibility of overnight communication with the subjects was established vocally through a microphone-speaker console system. Upon awakening, all subjects reported to have had a comfortable and undisturbed sleep, as also verified by measuring the relevant polygraphic parameters (see Kokkinos and Kostopoulos, 2011). Subjects that exhibited inadequate quality of sleep, due to the first-night effect, were excluded from this study.

All procedures described were approved by the Ethics Committee of the Medical School of University of Patras.

### Recording

Night sleep was recorded using 58 EEG tin electrodes according to the extended international 10–20 system (FP_1_, FP_Z_, FP_2_, AF_3_, AF_4_, F_7_, F_5_, F_3_, F_1_, F_Z_, F_2_, F_4_, F_6_, F_8_, FC_5_, FC_3_, FC_1_, FC_Z_, FC_2_, FC_4_, FC_6_, T_7_, C_5_, C_3_, C_1_, C_Z_, C_2_, C_4_, C_6_, T_8_, TP_7_, CP_5_, CP _3_, CP _1_, CP_Z_, CP _2_, CP _4_, CP_6_, TP_8_, P_7_, P_5_, P_3_, P_1_, P_Z_, P_2_, P_4_, P_6_, P_8_, PO_7_, PO_3_, PO_1_, PO_Z_, PO_2_, PO_4_, PO_8_, O_1_, O_Z_, O_2_) using an electrode cap (ElectroCap International Inc., Eaton, OH, USA). The EEG electrode inputs were linked-ear referenced and grounded over the F_Z_A position. A bipolar derivation of oblique EOG was used to detect eye-movements, for which electrodes were placed 1 cm above the right outer canthus and 1 cm below the left outer canthus. A bipolar EMG from the upper masseter muscle was used to track muscle tone changes. Impedance of all electrodes was kept below 10 k Ohms for most of the night. All electrophysiological parameters were AC recorded, amplified at a total gain of 1000, band-pass filtered at 0.05–500 Hz, and digitized through an 16 bit-resolution A/D converter, providing an accuracy of 0.084 uV/LSB, at a sampling frequency of 2500 Hz by a Synamps system (Neuroscan Inc., Charlotte, NC, USA), and stored on hard disk. The notch filter of 50 Hz was not applied during recording. Subject movements during sleep were detected by a sensitive motion-detector placed over the bed area that produced 2 s transistor-transistor logic (TTL) signal every time movement occurred. The motion-detector's signal was recorded as an external trigger and was stored along with the electrophysiological signals on a separate event channel.

### Scoring and event selection

**Figure 1:**
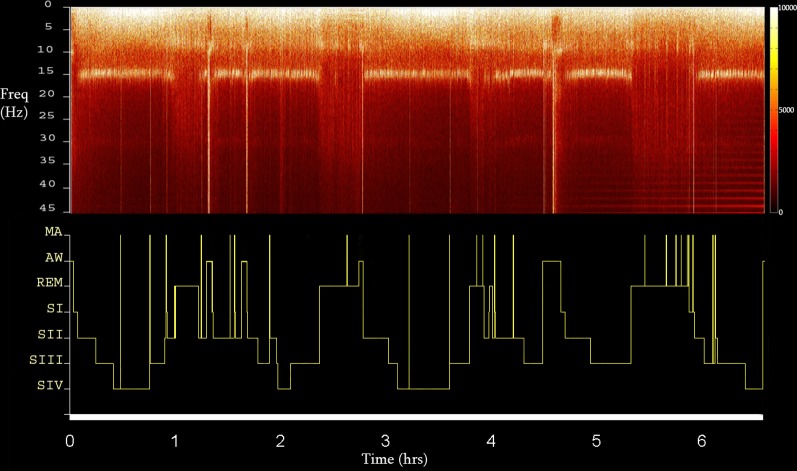
**The FFT-based whole Hypnospectrogram derived from the Cz electrode (upper) and the respective Hypnogram (lower) (MA, microarousal; AW, awake; REM, rapid eye-movement sleep; SI-IV, NREM sleep stages 1–4) of Subject 2.** Color power bar units are in uV^2^.

Sleep staging was performed by visual inspection according to standard criteria of Rechtschaffen and Kales (1968), taking under consideration the propositions of the AASM Visual Scoring Task Force (2007) as well as those of the DGSM Task Force (2006). Scoring was further assisted by the FFT-based (Fast Fourier Transform) hypnospectrogram that is, the whole-night FFT-based time-frequency plot for 0.05–45 Hz with a step frequency of 0.05 Hz (Kokkinos et al., 2009) (Figure 1). Microarousals were scored according to the guidelines of the ASDA Report (1992).

**Figure 2:**
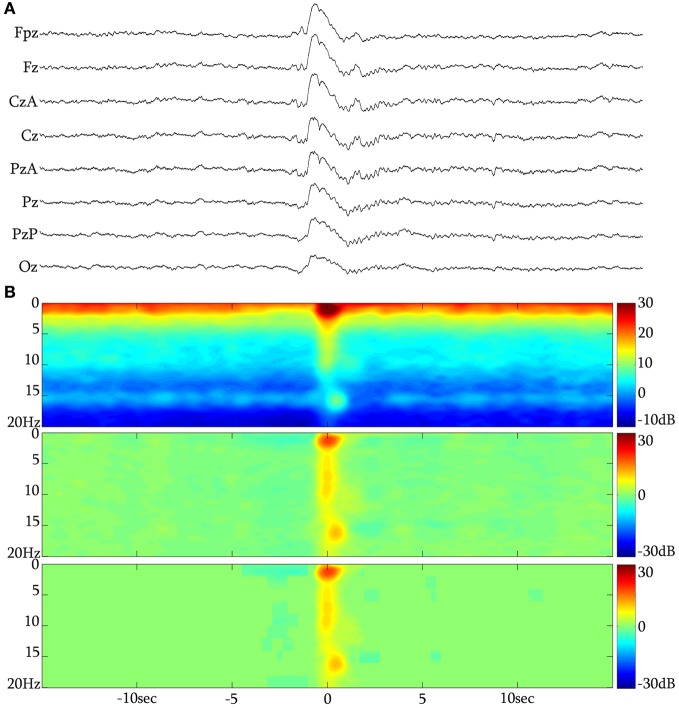
**(A)** The KC over the NREM background. **(B)** Long time-range average spectrogram (up), event-related spectral perturbation (middle) and statistically significant changes with respect to a −15 to −5 s background (low) in triplets for a grand-average of 313 unclustered KCs from subject 1.

**Figure 3:**
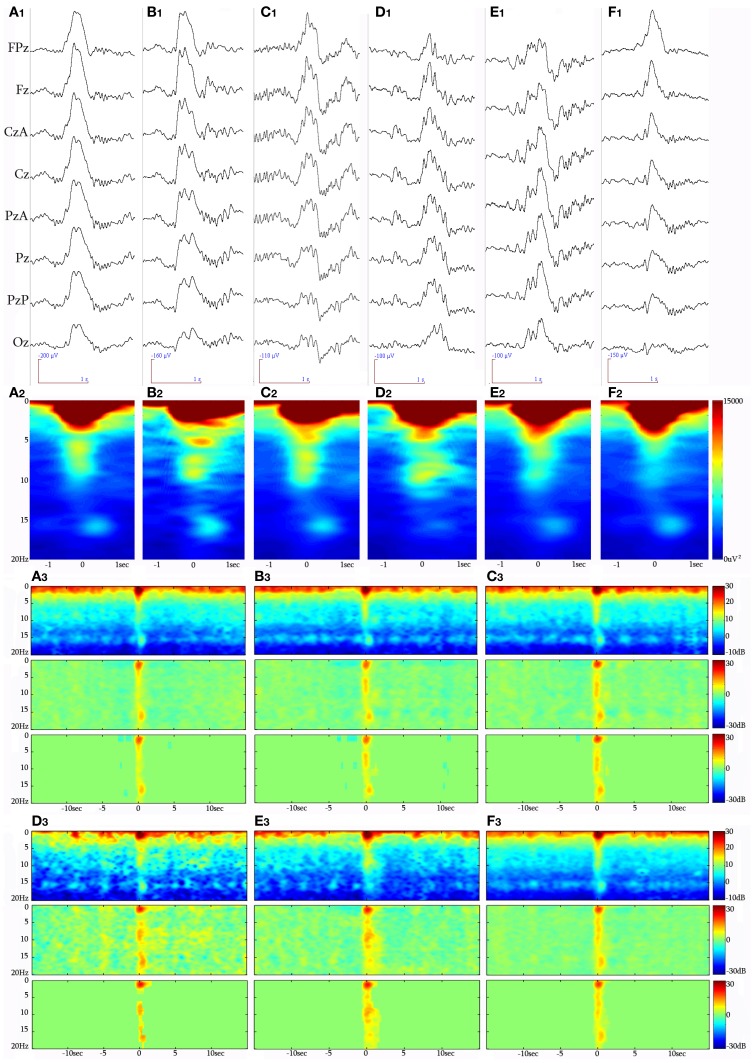
**Raw EEG (8 synchronous central derivations) samples of KCs identified during NREM sleep periods of different participants, forming the clustered groups analyzed in this study. (A_1_)** KC with a 2-peak iKCo during its electro-negative phase, **(B_1_)** KC with 3-peak iKCo, **(C_1_)** KC with 4-peak rhythmic iKC activity, **(D_1_)** KC crowned by alpha oscillations, **(E_1_)** KC with non-rhythmic activity during its negative phase, and **(F_1_)** KC without oscillatory activity during the course of its negative phase. Below (**A_2_–F_2_**) appear the respective spectral averages from the samples of each KC cluster. Long-time-range average spectrogram (up), event-related spectral perturbation (middle) and statistically significant changes with respect to a −15 to −5 s background (low) in triplets for each KC cluster (**A_3_** for non-iKCo, **B_3_** for 2-peak iKCos, **C_3_** for 3-peak iKCos, **D_3_** for 4-peak iKCos, **E_3_** for alpha-KCs and **F_3_** for non-oscillatory KC activity).

Selection of KCs was performed by all authors. The selected events were in turn forwarded for analysis after unanimous consensus. The KC was identified as a >500 ms well-delineated negative sharp wave that stands out of the EEG background, usually followed by a positive phase (Figure 2A). In this study, singular (without another KC or slow wave activity immediately preceding or following) generalized (distinguishable in the EEG all across the midline electrodes) spontaneously occurring KCs during NREM stages II and III were selected. KCs immediately preceding microarousals and awakenings during sleep, as well as KCs followed by delta waves, were excluded from this study. KCs were divided in three major categories: (1) KCs without intra-KC-activity (iKCa) over their negative phase, (2) KCs with iKCa that, by visual estimation, had no oscillatory features, and (3) KCs with oscillatory iKCa (intra-KC-oscillation, briefly iKCo). KCs with iKCos were further categorized as: (1) KCs incorporating 2 electro-negative peaks of clear oscillatory activity around their negative peak (Figure 3A_1_), (2) KCs incorporating 3 electro-negative peaks of clear oscillatory activity around their negative peak (Figure 3B_1_), and (3) KCs incorporating 4 electro-negative peaks of clear oscillatory activity around their negative peak (Figure 3C_1_), (4) KCs covered by alpha rhythm during their time-course (Figure 3D_1_). KCs that (1) did have iKCa appearing around their negative peak but its features were not oscillatory (incorporating asymmetric, discontinuous and non-sinusoidal features) (Figure 3E_1_), and (2) did not incorporate any oscillation or other activity during their course (Figure 3F_1_), were not included in the analysis that followed, as the asymmetrical morphology of the oscillation as well as its absence from the KC rendered them unlikely to fit the analysis used in this study. In this above categorization, sleep spindle activity following or preceding the KC was ruled out as a featured oscillation since it is not the objective of this study.

### Analysis

**Figure 4:**
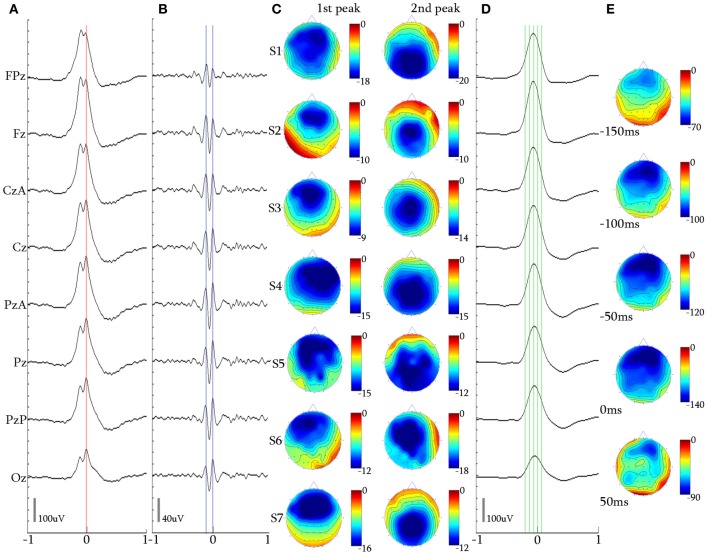
**2-peak iKCo. (A)** Waveform averaged KC—unfiltered EEG signal of subject 1. **(B)** 5 Hz high-pass filtered waveform of the averaged KC signal of the same subject. It can be noted that the first and second waves have maximal power in the frontal and occipital leads, respectively. **(C)** Topographic analysis in 2D electrode space for all subjects (S1–S7) of the 1st and 2nd peaks of the iKCo. The first wave in all subjects was maximum frontally and the second either parietally or occipitally. **(D)** 4 Hz low-pass filtered waveform of the averaged KC signal of subject 1. **(E)** Topographic analysis in 2D electrode space of the averaged slow KC wave, representing the KCs that contained 2-peak iKCo of subject 1 at the times marked by the green cursor in **(D)** [Red cursor: marked reference of the 2nd peak for waveform averaging, Blue cursors: marked peaks for the topographic analysis in **(C)**], Color bar units are in uV. Horizontal axis units of **(A,B**, and **D)** are in seconds.

**Figure 5:**
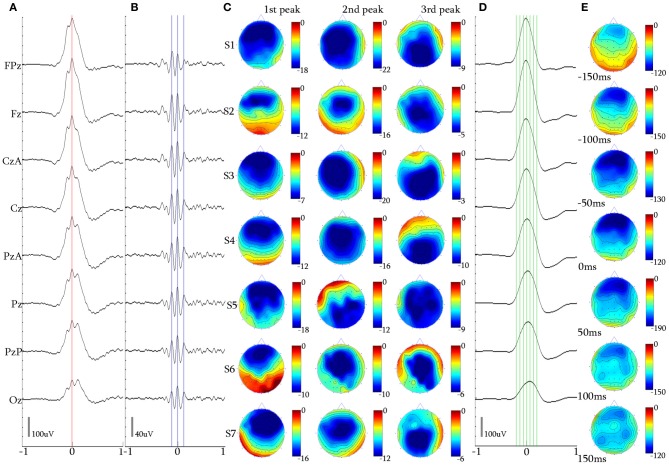
**3-peak iKCo. (A)** Waveform averaged KC—unfiltered EEG signal of subject 1. **(B)** 5 Hz high-pass filtered waveform of the averaged KC signal, of the same subject. **(C)** Topographic analysis in 2D electrode space for all subjects (S1–S7) of the 1st, 2nd, and 3rd peaks of the iKCo. Note than maximal power of the first, second and third wave was, respectively, located in frontal, central and parietal areas—with the exception of subject 5 where it was frontal-parietal-central. **(D)** 4 Hz low-pass filtered waveform of the averaged KC signal, of subject 1 at the times marked by the green cursor in **(D)**. **(E)** Topographic analysis in 2D electrode space of the averaged slow KC wave, representing the KCs that contained 3-peak iKCo (cursors and axis as in Figure 4).

**Figure 6:**
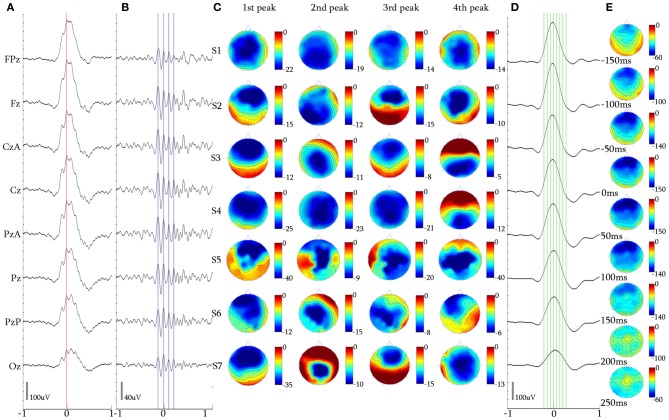
**4-peak iKCo. (A)** Waveform averaged KC signal—unfiltered EEG signal of subject 3. **(B)** 5 Hz high-pass filtered waveform of the averaged KC signal. **(C)** Topographic analysis in 2D electrode space for all subjects (S1–S7) of the 1st, 2nd, 3rd, and 4th peaks of the iKCo. **(D)** 4 Hz low-pass filtered waveform of the averaged KC signal. **(E)** Topographic analysis in 2D electrode space of the averaged slow KC wave, representing the KCs that contained 4-peak iKCo (cursors and axis as in Figure 4).

**Figure 7:**
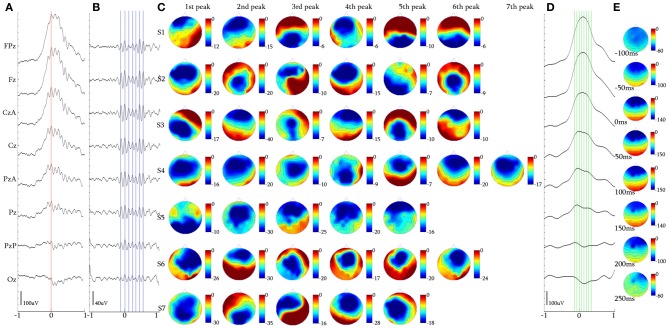
**Alpha KCs. (A)** Waveform averaged KC signal—unfiltered EEG signal of subject 4. **(B)** 5 Hz high-pass filtered waveform of the averaged KC signal. **(C)** Topographic analysis in 2D electrode space for all subjects (S1–S7) of the 1st–7th peak of the alpha oscillation. **(D)** 4 Hz low-pass filtered waveform of the averaged KC signal. **(E)** Topographic analysis in 2D electrode space of the averaged slow KC wave, representing the KCs crowned with alpha waves (cursors and axis as in Figure 4).

Event marking was performed manually by Scan software (Neuroscan Inc., Charlotte, NC, USA) in order to create event channels. NREM stage II and III epochs from the whole-night sleep recording were selected and precise time-markers were placed over the events under study. In total, four kinds of events were visually marked and further used for referenced analysis, as explained below: (1) the second electro-negative peak of the 2-peak iKCo on the negative phase of the KC (Figure 4A), (2) the second electro-negative peak of the 3-peak iKCo on the negative phase of the KC (Figure 5A), (3) the second electro-negative peak of the 4-peak iKCo on the negative phase of the KC (Figure 6A), and (4) the second electro-negative peak of the alpha-crowned KC (Figure 7A). For all cases we marked the time of the signal peak in the electrode of maximal amplitude.

Event-related data were further processed by a custom-made Matlab-based (The Mathworks, Natic, MA, USA) software suite developed at the Neurophysiology Unit. Each of the selected NREM KCs underwent two separate filterings by means of a finite impulse response (FIR) least squares filter (Oppenheim and Schäfer, 1989) in order for two clusters to be created: (1) a cluster of data incorporating the oscillatory component by high-pass filtering at 5 Hz (double-pass, zero-phase distortion, order 1500, −30 dB/oct) (see Figures 4B, 5B, 6B, and 7B), and (2) a second cluster of data incorporating the slow KC wave by low-pass filtering at 4 Hz (double-pass, zero-phase distortion, order 1875, −30 dB/oct) (see Figures 4D, 5D, 6D, and 7D). Scalp mapping of the recorded data was performed by the use of the Green-function-based multiquadric interpolation method described by Sandwell (1987), after baseline correction of all channels. In order for the duration of each electro-negative wave of the iKCo to be determined, the time interval (*t*) between the previous and the following electro-positive peak was measured from the high-pass 5 Hz filtered signal. Accordingly, the inferred instantaneous frequency of each wave was calculated as *f* = 1/*t*. The theta spectral EEG range is defined as the 5–8 Hz band and the alpha range as the 9–12 Hz band, with the 8–9 Hz range as the transition band.

FFT-based event-related time-frequency analysis (TFA) was performed by means of the short-time Fourier transformation, of a 4096-point window and a 4000-point overlap, thereby providing ~40 ms time bin. Statistical significance of patterns in time-frequency plots for Figures 3A_3_–F_3_ was determined by the method described by Zygierewicz et al. (2009). Time-frequency elements of 2 Hz frequency resolution and 0.25 s time resolution were calculated using the corresponding mean spectral values. In turn, the Box-Cox transformation was used to transform the values across events to an approximate normal distribution. The null hypothesis of no change from a baseline period −15 to −5 s prior to the event marker was tested for each element using *t*-test, assuming unequal variances (Welch *t*-test). Correction for multiple comparisons was performed by controlling false discovery rate with *q* = 0.05 (Durka et al., 2004). Relative changes of spectral power were calculated using the ratio of the original mean values of the power spectral density for every time-frequency bin to the average of the values during the baseline period (Pfurtscheller and Lopes da Silva, 1999), and the logarithm of this ratio was plotted for significant patterns. For the TFA averaged plots of individually transformed trials, the mean power of the baseline period is used as the denominator in the logarithmic dB scale. For the non-normalized plots, the denominator corresponds to the power spectral density of 1 uV^2^.

## Results

In total, 1522 KCs were identified and selected from NREM II and III stages of all sleep cycles of the seven participants. Of those, 1229 (80.75%) had iKCa over their negative phase, and 293 (19.25%) had no apparent activity whatsoever during their negative phase peak (Figure 3F_1_). From the KCs with iKCa, 786 KCs (63.96%) had clear oscillation during their negative phase, 364 (29.62%) exhibited activity during their negative phase that had no clearly organized oscillatory features (and therefore could not be processed with averaging methods) (Figure 3E_1_), and 79 (6.42%) were accompanied by alpha activity either before, during or after their negative phase (Figure 3D_1_ shows a sample of alpha rhythm during the KC's negative phase). Out of 786 KCs that had clear oscillatory activity during their negative phase, 386 (49.10%) had only 2 consecutive peaks (Figure 3A_1_), 344 (43.76%) had only three consecutive peaks (Figure 3B_1_), and 56 (7.12%) had only four peaks (Figure 3C_1_). Table 1 summarizes the KC clusters per subject. Respectively, the KCs incorporating oscillatory activity of 2 peaks had mean duration 603.42 ± 65.07 ms, those incorporating 3-peak oscillations had mean duration 694.28 ± 52.81 ms, and KCs incorporating oscillation of 4 peaks had mean duration of 742.42 ± 96.77 ms. Averaged TFA of ±1.5 s around the KCs negative peak (Figures 3A_2_–F_2_), for each of the above categories, justified the morphological time-dimension categorization as each KC cluster exhibits distinctive spectral features corresponding to the visual EEG observations. There are noticeable differences in frequency range of the iKCo for the 3 clusters identified (2-, 3-, and 4-peak oscillations in Figures 3A_2_–C_2_, respectively), whose spectral profile in the 5–12 Hz range is clearly distinct from both KCs with alpha waves (Figure 3D_2_) and KCs lacking iKCo (Figure 3F_2_). iKCos of non-oscillatory character over the negative KC peaks exhibit a less clear spectral distribution (Figure 3E_2_) but differs for the others. Long-term averaged spectral analysis ±15 s around the KCs (Figures 3A_3_–F_3_, first of each triplet), as well as the respective statistical spectral plots (Figures 3A_3_–F_3_, second and third of each triplet) show that the iKCo is a statistically significant phenomenon with respect to the NREM background activity surrounding the KC. The latter is also supported by grand-averaging KCs (Figure 2B).

**Table 1:** **Group percentages of the number of KCs identified during the whole-night sleep of the participants**.

	**Subject 1**	**Subject 2**	**Subject 3**	**Subject 4**	**Subject 5**	**Subject 6**	**Subject 7**	**Means**
2-peak iKCo	60 (19.23%)	42 (22.71%)	64 (25.20%)	25 (20.33%)	43 (22.27%)	85 (36.96%)	67 (29.91%)	386 (25.36%)
3-peak iKCo	72 (23.07%)	42 (22.71%)	65 (25.60%)	37 (30.09%)	47 (24.36%)	53 (23.05%)	28 (12.50%)	344 (22.61%)
4-peak iKCo	11 (3.52%)	4 (2.16%)	20 (7.87%)	10 (8.13%)	1 (0.51%)	7 (3.04%)	3 (1.34%)	56 (3.67%)
iKC alpha	24 (7.68%)	11 (5.94%)	7 (2.75%)	9 (7.31%)	17 (8.81%)	8 (3.47%)	3 (1.34%)	79 (5.19%)
KCs with non-rhythmic activity	81 (25.95%)	48 (25.94%)	38 (14.96%)	31 (25.20%)	58 (30.06%)	43 (18.70%)	65 (29.01%)	364 (23.92%)
KCs without oscillation	65 (20.82%)	38 (20.54%)	60 (23.62%)	11 (8.94%)	27 (13.99%)	34 (14.78%)	58 (25.90%)	293 (19.25%)
Total KCs	313	185	254	123	193	230	224	1522

*Means appear on the last column*.

### The 2-peak intra-KC oscillation

Topography over the waveform averaged 2-peak iKCo (Figures 4A,B) showed that in 90.15% (348/386) of KCs: (1) the first peak had frontal distribution (Fz level) in 7/7 of the participants, (2) the second peak had posterior distribution shift toward the central (Cz level), in 3/7 of participants and parietal areas (Pz level), in 4/7 of participants (compare Figures 4C–D for each subject). In the rest of the samples (38/386, 9.85%) the observed pattern of propagation was reversed that is, the oscillation followed a posterio-anterior shift pattern. In Figures 4D,E, the waveform and time-course of the slow wave in electrode space can be seen, respectively, where one can easily observe that: (1) the maximum of the negative peak of the KC lies frontally, as is already well-documented in literature (Colrain, 2005), and (2) there are no indications for shifting of the maximum of the negative peak toward other cortical areas. This analysis showed that on average the KC does not follow the average propagation pattern of the iKCo.

For the majority of 2-peak iKCos that exhibited the anterio-posterior pattern of spatial shift (348/386, 90.15%), the mean duration for all subjects of the first wave of the iKCo was found to be 134.85 ± 9.89 ms, which corresponds to an instantaneous frequency of 7.44 ± 0.53 Hz. The mean duration for all subjects of the second wave of the iKCo was found to be 138.57 ± 9.07 ms (7.24 ± 0.46 Hz). These mean durations were not significantly different (*p* = 0.478, *t* = −0.73, *df* = 12, 2-tailed paired *t*-test). For the minority of 2-peak iKCos that exhibited the posterio-anterior pattern (38/386, 9.85%), the mean duration for all subjects of the first wave of the iKCo was found to be 137.22 ± 17.70 ms (7.37 ± 0.96 Hz), and the mean duration for all subjects of the second wave of the iKCo was found to be 136.54 ± 14.30 ms (7.34 ± 0.77 Hz). These mean durations were also not significantly different (*p* = 0.987, *t* = 0.01, *df* = 10, 2-tailed paired *t*-test).

### The 3-peak intra-KC oscillation

Topography of the averaged 5 Hz high-pass filtered 3-peak iKCos (Figures 5A,B) in 2D electrode space showed that in 89.54% (308/344) of KCs: (1) the first peak of the iKCo had frontal distribution (Fz level) in 7/7 of subjects, (3) the second peak had central distribution (Cz level) in 6/7 of participants; in one participant (1/7, S5 in Figure 5C) the second peak had parietal distribution (Pz level), and (3) the third iKC peak had parietal distribution (Pz and PzA level) in 6/7 of participants; the same subject whose second peak lay parietally (1/7, S5 in Figure 5C), had a central distribution for the third iKC peak (Cz level). In all participants, except one, the iKCo exhibited a clear anterio-posterior pattern of distribution; the one in exception showed initially a pattern of anterio-posterior shift and in turn an opposite posterio-anterior shift (Figure 5C). In this cluster of data as well, a small percentage of samples (36/344, 10.46%) had iKCo that followed a posterio-anterior pattern of propagation. The results of the topographic analysis for the slow KC wave (Figures 5D,E) were the same as previously, showing maximal frontal distribution, without elements of cortical propagation.

The mean duration for the majority of 3-peak iKCos that exhibited the anterio-posterior spatial shift pattern (308/344, 89.54%) in all subjects of the first iKCo wave was found to be 130.57 ± 9.88 ms (7.69 ± 0.57 Hz). The mean duration for all subjects of the second iKCo wave was found 111.14 ± 10.22 ms (9.06 ± 0.87 Hz), and the mean duration for all subjects of the third iKCo wave was found to be 143.28 ± 13.27 ms (7.03 ± 0.70 Hz). For all subjects the mean duration of the second wave was significantly shorter than that of the first wave (*p* = 0.003, *t* = −3.61, *df* = 12, 2-tailed paired *t*-test) and the mean duration of the third wave was significantly longer than that of the second wave (*p* = 0.000, *t* = −5.07, *df* = 11, 2-tailed paired *t*-test), while the first and third had no statistically significant difference in duration (*p* = 0.066, *t* = −2.03, *df* = 11, 2-tailed paired *t*-test). For the minority of 3-peak iKCos that exhibited the posterio-anterior pattern (36/344, 10.46%) and for the 4/7 subjects (S3, S5–S7) in whose sleep they were identified, the mean duration of the first wave of the iKCo was found to be 141.50 ± 11.35 ms (7.07 ± 0.52 Hz), the mean duration of the second wave of the iKCo was 105.51 ± 5.80 ms (9.49 ± 0.51 Hz), and the mean duration of the third wave was 130.75 ± 7.78 ms (7.66 ± 0.77 Hz). For this minority of samples as well, the mean duration of the second wave was significantly shorter than that of the first wave (*p* = 0.004, *t* = 5.64, *df* = 4, 2-tailed paired *t*-test) and the mean duration of the third wave was significantly longer than that of the second wave (*p* = 0.023, *t* = −3.57, *df* = 4, 2-tailed paired *t*-test); the first and the third wave did not exhibit statistically significant differences in duration (*p* = 0.257, *t* = 1.25, *df* = 6, 2-tailed paired *t*-test). The instantaneous frequency is thereby shown to be initially increased and subsequently decreased in all samples, independent from their topographical diversity.

### The 4-peak intra-KC oscillation

The 2D topographic analysis in 52/56 (92.85%) of KC samples incorporating 4 peaks of iKCo (Figures 6A–C) showed that: (1) the first peak had frontal distribution (Fz level) in 6/7 of subjects and central distribution in one (1/7) of the participants, (2) the second peak had parietal distribution (PzA and Pz level) in 4/7 of subjects and central distribution (CzA and Cz level) in 3/7 of subjects, (3) the third peak had frontal distribution (Fz level) in 4/7 of participants, in 2/7 had parietal distribution (Pz level) and in 1/7 had central distribution (Cz level), and (4) the fourth peak had central distribution (Cz level) in 3/7 of participants, parietal distribution (Pz and POz level) in 3/7 of participants and frontal (Fz level) in 1/7 of participants. The general view of the 4-peak iKCo reveals recurrent shifts of maxima between frontal, central and parietal areas (Figure 6C), clearly with a lesser degree of pattern reproducibility among subjects (although each topographic pattern was quite robust for each subject separately). In the case of 4-peak iKCo as well, in a small percentage of samples (4/56, 7.14%) the identified topographical pattern begun inversely over the parietal areas and in turn shifted anteriorly with reciprocal relocation of the amplitude maxima. The topographic analysis for the slow KC wave showed again maximal distribution over the frontal areas which lacked elements of propagation or shift toward extra-frontal areas (Figures 6D,E).

For the majority of 4-peak iKCos that exhibited an anterior onset (52/56, 92.85%), the mean duration of the first iKCo wave was found to be 123.51 ± 9.29 ms (8.13 ± 0.58 Hz). The mean duration for all subjects of the second iKCo wave was found 108.52 ± 13.10 ms (9.31 ± 1.00 Hz), the mean duration for all subjects of the third iKCo wave was found to be 105.21 ± 9.90 ms (9.57 ± 0.86 Hz), and the mean duration for all subjects of the fourth iKCo wave was found to be 110.60 ± 10.88 ms (9.11 ± 0.91 Hz). For all subjects the mean durations of the second, the third and the fourth wave did not significantly differ from each other (2nd–3rd: *p* = 0.422, *t* = 0.83, *df* = 10; 3rd–4th: *p* = 0.534, *t* = −0.64, *df* = 10; 2nd–4th: *p* = 0.775, *t* = 0.29, *df* = 9, 2-tailed paired *t*-test) but were all significantly shorter than the duration of the first wave (1st–2nd: *p* = 0.046, *t* = 1.87, *df* = 9; 1st–3rd: *p* = 0.011, *t* = 3.11, *df* = 10; 1st–4th: *p* = 0.028, *t* = 2.55, *df* = 10, 2-tailed paired *t*-test). For the minority of 4-peak iKCos that exhibited a posterior onset (4/56, 7.14%) in 2/7 subjects (S3 and S4), the mean duration of the first iKCo wave was found to be 138.54 ± 7.77 ms (7.22 ± 0.41 Hz), the mean duration of the second iKCo wave was found 106.48 ± 2.12 ms (9.31 ± 0.19 Hz), the mean duration of the third iKCo wave was found to be 108.00 ± 8.48 ms (9.24 ± 0.73 Hz), and the mean duration of the fourth iKCo wave was found to be 129.55 ± 14.84 ms (7.75 ± 0.87 Hz).

### Alpha rhythm over KCs

Upon selection of KCs that incorporated alpha rhythmic activity, we identified three main categories: (1) KCs where alpha oscillation appeared before its rising negative edge and was already terminated around its peak (15/79, 18.99%), (2) KCs where alpha covered its negative peak from the rising edge to the falling edge (22/79, 27.85%), and (3) KCs where alpha followed the falling edge of the KC and continued oscillating after the KCs passing (42/79, 53.16%). In the context of this study, where the behavior of oscillatory activity during the negative peak of the KC is the objective, only the second sub-group of KCs was selected for analysis, despite the fact that the majority in this cluster comprised KCs with post-KC alpha activity. This sub-group, where the alpha oscillation was distributed over the negative peak of the KC (see Figure 3D_1_), was used as control activity for the iKCo investigated both in terms of spatial and spectral distribution.

Topographical analysis of KCs that incorporated alpha oscillation over their negative phase (Figures 7A,B) showed that there was no systematic pattern of peak-after-peak spatial distribution in this sub-group of alpha KCs (see Figure 7C). Nevertheless, the analysis showed that alpha waves do not maximize over the same electrode space location with every peak but rather follow a variable pattern of spatial shift along the anterio-posterior axis. The results of the topographic analysis for the slow KC wave (Figure 7D) showed maximal frontal distribution without spatial shifting (Figure 7E). The mean number of alpha waves over the KC for every subject varied from 5 to 7 waves.

The mean duration of the first alpha wave was 113.85 ± 7.02 ms (8.81 ± 0.54 Hz), of the second was 104.82 ± 1.12 ms (9.63 ± 1.05 Hz), of the third 91.61 ± 4.49 ms (10.93 ± 0.54 Hz), of the fourth 104.91 ± 9.10 ms (9.59 ± 0.92 Hz) and of the fifth wave was 105.47 ± 13.85 ms (9.62 ± 1.31 Hz). From 5/7 subjects that had 6-wave alpha, the mean duration of the sixth wave was 110.60 ± 10.99 ms (9.12 ± 1.03 Hz), and from 1/7 that had 7-wave alpha the duration of the seventh wave was 104.70 ms (9.55 Hz). The alpha waves varied within the alpha band limits, with their instantaneous frequency exhibiting a tendency to be initially increased toward upper alpha band limits and subsequently decreased toward the lower alpha band limits regardless of their significant topographical variability.

### The iKCo and KC-alpha spectral profiles

**Figure 8:**
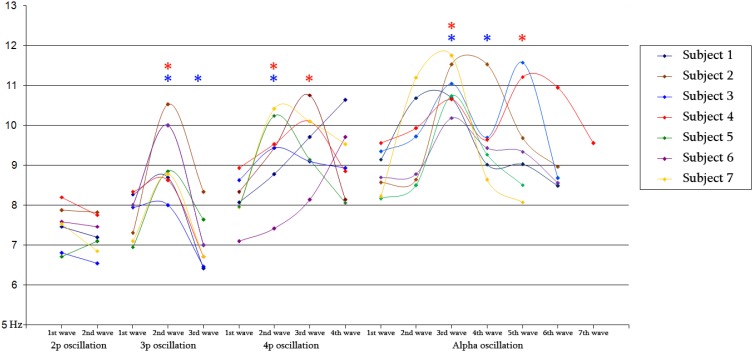
**Diagram of mean frequency values for 2-peak (*N* = 348/386), 3-peak (*N* = 308/344) and 4-peak (*N* = 52/56) iKC oscillations, as well as for iKC alpha rhythmic activity, as they appear on Table 2, for all subjects.** Blue and red stars denote a statistically significant change of mean instantaneous frequency compared to that of the preceding wave and that of the 1st wave, respectively.

The individual instantaneous frequency values for each wave of the 4 clusters of iKCo identified in this work for every subject appear in Figure 8. The instantaneous frequencies of the waves comprising the 2-peak iKCo both remain within the theta band. Regarding the 3-peak iKCo, the frequency of the first wave lies within the limits of the theta band, the second enters the alpha band and the third returns to the initial theta band levels. As for the 4-peak iKCo, its first wave is already at the lower limits of the alpha band, and the two waves that follow (2nd and 3rd) are within the alpha band limits. The fourth wave, in 4/6 of subjects, tended to return to the initial frequency values at the lower end of the alpha band, whereas in 2/6 subjects the fourth wave tended to further increase in frequency toward the upper limits of the alpha band. Regarding the identified alpha rhythmic activity as control, each of the consecutive waves of the oscillation remained within the alpha band limits, with an observable tendency of the intermediate waves to escalate spectrally toward the upper alpha band and the late waves to return to the initial lower alpha levels.

## Discussion

This study aimed to determine the features of the short-lived oscillation in the 7–9 Hz range observed over the negative peak of NREM KCs, a phenomenon occurring in almost 3/4 of KCs. Theta waves do appear in the NREM II and III background (Rechtschaffen and Kales, 1968), and the A-phases of the cyclic alternating pattern (CAP) (Terzano et al., 1985). They are less often observed in oscillatory fashion and wide distribution over the NREM background. Alpha oscillations do appear in NREM, mainly during CAP-A phases, less often during CAP-B NREM phases, and usually represent transient fluctuations of the level of vigilance (Halász, 1993). The present study was based on the observation (by visual investigation of the EEG) that rhythmic and non-rhythmic activity of the theta-alpha range and of prominent amplitude appears systematically over the negative phase of the majority of singular KCs. Although theta waves and oscillations do appear sparsely and randomly distributed over the NREM background, they do not systematically appear in the immediate NREM background surrounding the KC. The latter (i.e., the specificity of appearance of theta oscillation only during the negative phase of KCs) was supported by spectral and statistical spectral analysis of the averaged clustered samples (Figures 3A_3_–F_3_). We identified and selected all the NREM II and III KCs. We indentified three major categories: KCs without iKCa, KCs with iKCa bearing no promonent oscillatory features, and KCs in which we observed rhythmic activity (iKCo) during their negative phase. From the total volume of KCs with iKCo, we created four clusters for further analysis: KCs with iKCo of 2 peaks, KCs with iKCo of 3 peaks, KCs with 4 peaks of oscillation during their electro-negative part, and KCs with alpha activity over their negative phase. The three former clusters corresponded to KCs of increasing duration. We set as reference point the second peak of each oscillation in each of the four KC groups. In turn, event-related signal analysis was performed, from which waveform averages and topographic plots were created. At the same time, changes in oscillation instantaneous frequency were investigated, by measuring for each electro-negative intra-KC peak the distance between the adjacent electro-positive peaks, thereby deriving the duration of each electro-negative wave.

The topographic analysis of the iKCo (Figures 4–7 and Table 2) showed that: (1) the 2-peak and 3-peak iKCo follow an anterio-posterior direction pattern of cortical propagation of maximal power in 2D electrode space, (2) the 4-peak iKCo is following a recurrent pattern of propagation over the anterio-posterior axis, (3) the alpha KC activity is not following systematic pattern, which would incorporate peak maximization shifts over the anterio-posterior axis, and (4) the propagation pattern of the slow KC wave follows neither those of the iKCo nor those of the alpha rhythm. It is important to note here that for each of the 3 iKCo categories, a small proportion of samples exhibited opposite propagation patterns of posterio-anterior direction. This fact shows that onset and direction of iKCos may vary within subjects, but still maintain a separate spatial distribution profile than that of the slow KC wave.

**Table 2:**
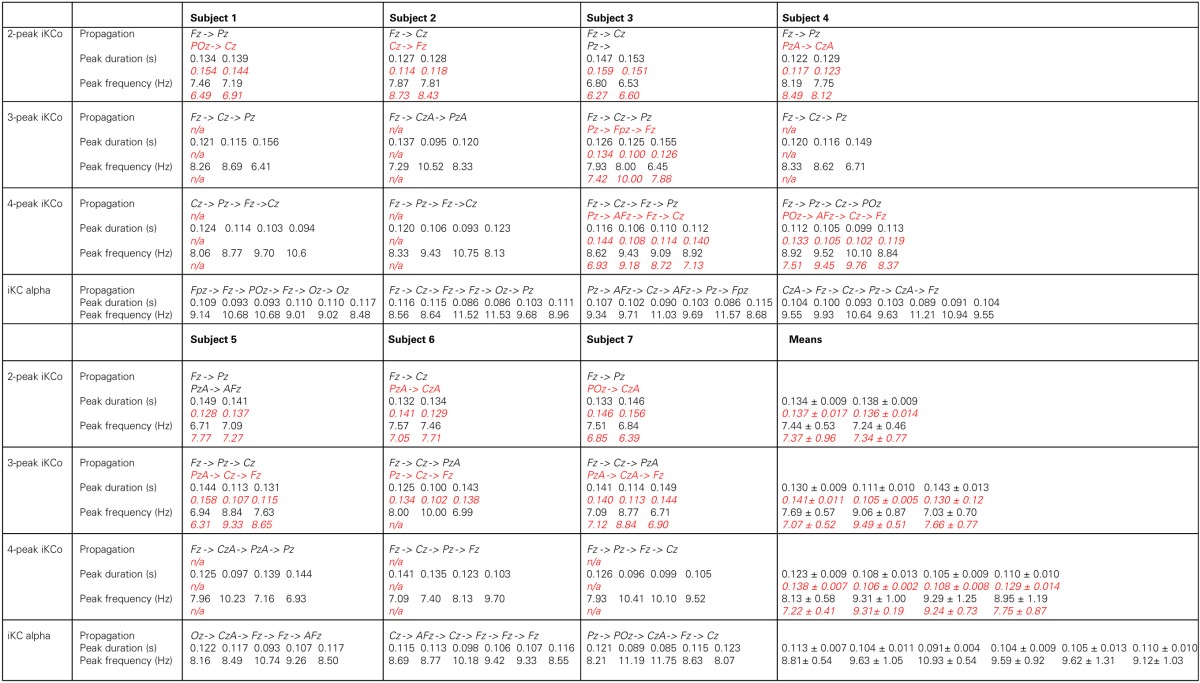
**Topographical and numerical results of direction of propagation, wave duration and inferred frequency for each wave of the averaged and high-pass filtered 2-peak, 3-peak, 4-peak iKCo, and iKC alpha, for each subject, where applicable, in black**.

*In red, appear the same results for KC populations that exhibited inverse topographical shift patterns. Mean values ± SD appear on the last column*.

The analysis of frequency features of each wave of the iKCo (Figure 8 and Table 2), with minor exceptions, showed that: (1) for the 2-peak iKCo, both waves tend to remain within the theta band limits, tending to occupy the upper portion of the band, (2) for the 3-peak iKCo, the first wave lies at the upper limit of the theta band, the second crosses to the alpha band, and the third returns to the initial levels of the theta band, (3) for the 4-peak iKCo, the first wave is already at the lower limits of the alpha band, the second and the third wave clearly reside within the limits of the alpha band and the fourth wave, most often, tends to return toward the initial low alpha band levels. The control KC group incorporated consecutive waves that occupied spectrally the alpha band and, similar to the 2-, 3-, and 4-peak iKCos, exhibited a pattern with the intermediate waves shifting their instantaneous frequency toward the upper limits of the alpha band and the late waves shifting spectrally toward the lower alpha limits.

The possibility that the simple high pass filtering employed (>5 Hz) has contributed to the variation of amplitude and intervals of individual peaks in the iKCo can be excluded, since: (1) The same filtering applied to KCs devoid of iKCo (data not shown) expectedly results in much smaller peaks which all have maxima frontally like the slow negative KC wave and unlike the variable distribution of maxima of the filtered KCs with iKCo; which therefore can be considered as independent of the slow negative wave of KC. (2) If the localization of the phenomenon under study had a systematic and significant bias from the filtering artifact, the variability in topography especially observed in the 4-peak iKCos, would not appear. (3) The representative samples presented in Figure 3, as well as the waveform averages of Figures 4–7, show that the iKCo and its localization can be visually estimated in raw EEG. The analysis methods applied in this study are a simple means of highlighting, presenting and verifying what is already visually observed. In addition, averaging with reference point the second peak was expected to present the first and third waves relatively smaller than the second due any interval variation between single events. Apparently this variation in intervals was negligible compared to the observed tendency to fronto-occipitally relocate the maxima of the waves constituting the iKCo. This indicates that iKCo is a robust phenomenon repeating itself regularly throughout the night.

The topographic analysis therefore clearly showed that the iKCo under study does not constitute part of the slow KC wave, as the former follows individual propagation patterns in electrode space and thereby must be independent from the KC (at the same time justifying the term “complex” to describe this NREM EEG phenomenon). Massimini et al. (2004) showed that the slow (<1 Hz) NREM waves (including KC s and NREM delta waves) are individually travelling waves originating mainly anteriorly and shifting in the anterio-posterior axis. Yet, the grand average localization was frontal without a consistent pattern of cortical propagation, thereby verifying our negative results regarding the average topographical shift of the slow KC wave (see columns **D,E** in Figures 4–7). On the other hand, the iKCo showed systematic average shift of peak-after-peak maxima, thereby justifying the claim presented in this work of it being an independent component of sleep. In addition, the frequency analysis of each oscillation wave separately clearly showed that as the number of iKC waves is increased their instantaneous frequency has a tendency to shift from the theta band (a frequency range compatible with NREM sleep) toward the alpha band (incompatible with NREM sleep, although compatible with drowsiness, microarousals and REM sleep), with a late but also clear tendency of the late waves to have their instantaneous frequency within the frequency levels of the first wave (a short of dampening pattern, as if aiming in re-setting the frequency of the iKCo). The iKCo should not be considered as the intra-KC development of a sleep spindle that is degraded in frequency, for the following reasons: (1) there are KCs that contain iKCos without pre- or post-KC spindles, (2) there are KCs with both pre- and post-KC spindles without iKCo, (3) in KCs that iKCos and pre- and/or post-KC spindles occur there is no continuum between iKCo and spindles in either the time or the frequency domain, (4) the average topographical localization of the sleep spindle is stable over the posterior frontal and anterior parietal midline areas (Kokkinos and Kostopoulos, 2011).

To the best of our knowledge this is the first quantitative study of the described iKCo. Even though it has been illustrated clearly to occur during the negative phase of the slow wave in both spontaneous and evoked KCs (Ehrhart et al., 1981; Niiyama et al., 1996), it has not been the object of systematic research until now. The majority of studies dealing with the evoked KC average the signal with reference to the auditory stimulus (200–300 ms before the rising negative edge of the KC), thereby rendering any iKCo “invisible” by visual evaluation of the averaged results (Crowley et al., 2004; Nicholas et al., 2006). The visually evaluated and manually marked on raw EEG event-related methodology applied in this study proved to be most appropriate to reveal the features of this oscillatory activity, disassociating the latter from the slow wave of the KC and allowing its separate examination.

Earlier reports by Peter Halasz and colleagues that KCs express a double nature (Janus-face) (Colrain, 2005; Halász and Bodizs, 2013) are supported by recent fMRI evidence of sensory areas involvement in KCs (Caporro et al., 2012; Jahnke et al., 2012) and even the spontaneous KCs wear at their initial segment the traces of multisensory processing (Riedner et al., 2011). It is thus suggested that “K-complex embodies an arousal with subsequent sleep guarding counteraction that might on one hand serve monitoring of the environment with basic information processing and on the other hand protect continuity of sleep and thus its restoring effect” (Jahnke et al., 2012). According to this prevalent view, during KC the brain conducts low level cognitive processing to investigate the saliency or possible threat of external and internal stimuli and decides “not to wake up,” compensating the disturbing effect of the incoming stimulus by producing a KC. A role for KCs in sleep-specific endogenous information processing for external or internal stimuli has been proposed, based on works using “oddball” paradigm experiments in normal sleeping humans (Colrain et al., 1999). We tend to understand the mechanism of the sleep protective compensation with the help of older animal (Amzica and Steriade, 1997) and recent human (Cash et al., 2009) studies showing that the main negative phase of KC reflects a period of hyperpolarization (down state) of certain frontal areas, thus suppressing any arousal reactions to the non-threatening stimulus. However, we do not have any indication regarding the mechanisms of the low level cognitive processes. Perhaps our demonstration of EEG short oscillations associated in time with the KCs (iKCo) may provide hints toward such understanding. These iKCo appear to have features independent of the KCs, like a tendency to increase their spectral frequency from upper theta to low alpha as well as to move their maximal power fronto-occipitally.

The evidence of role of KC in memory consolidation is strong albeit indirect. There is ample evidence for a role in memory consolidation of stage two NREM, when KC appear—along with spindles (Stickgold, 2005; Rasch and Born, 2013). The KC amplitude and incidence are sensitive measures of normal healthy brain aging and in dementia due to alcoholism and Alzheimer disease the amplitude of KCs and their elicitability decreases (Colrain et al., 2009). Regarding the underlying mechanism, Cash et al. (2009) proposed that information processing during wakefulness lowers the activation thresholds of cortical synapses, making them more responsive, and so they need to be adjusted back in order to preserve their signal-to-noise ratio (synaptic homeostasis, Tononi and Cirelli, 2006). The down-state provided by KCs does this by reducing the strengths of synaptic connections that occur while an individual is awake (Cash et al., 2009). Further, the recovery from the down-state they induce allows that “cortical firing ‘reboots’ in a systematic order” so that memory engrams encoded during neuronal firing can be “repeatedly practiced and thus consolidated” (Cash et al., 2009). The observed features of the iKCo may prove useful markers in the search of further associations between KCs and cognitive processes or their deterioration with aging and disease.

The KCs have been also variously implicated in the pathophysiology of epileptogenesis, as well as seizure expression. Halász (1981) demonstrated manifold similarities between the KC and the spike-wave pattern of the EEG in generalized epilkepsy, which he regarded as an epileptic “caricature” of the sleep induction momentum reflected in the KC phenomenon. On the side of focal epilepsies, KC activity increases in nocturnal frontal lobe epilepsy, especially prior to a clinical seizure. According to Si et al. (2010), this reflects an unstable sleep condition, which suggests a correlation between KC and epileptic activities including seizures and EEG spike discharges. The observed features of the iKCo may prove useful markers in the search of further associations between KCs and manifestations and mechanisms of epilepsy.

Regarding the mechanisms generating iKCo, one should consider that the slow negative wave of a KC is proposed to represent an isolated cortical down state, i.e., a period of synaptic quiescence of cortical neurons (Cash et al., 2009). At the same time, however, sensory evoked responses are more easily produced during down as compared to up states, they propagate relatively further in cortex through recurrent excitation and they bias cortical neurons toward the up state (Petersen et al., 2003). Although the occurrence of KCs may be closely repeated and they can be grouped as in CAP activation (CAP-A) periods suggesting that they may be nested in, respectively, slow or even infra-slow cortical oscillations related to sleep instability, they often also occur as singular KCs, especially in the second NREM stage (Halász, 2005). In these cases their major surface-negative component constitutes a cortical down-state, i.e., a hyperpolarization of principal cortical neurons and a general decrease in cortical activity (see Cash et al., 2009 and also correspondence to this article). Animal experiments (Amzica and Steriade, 2002) and human studies (Colrain, 2005; Halász, 2005; Cash et al., 2009; Kohsaka et al., 2012), however, leave no doubt that KCs deserve their name since—especially when grouped—they also contain smaller depolarizing i.e., EEG surface-positive components (before and mainly after the major surface-negative one), while KCs elicitation is shown to be associated with both thalamic and brainstem activations and the appearance of spindles. The importance of understanding the apparently complex mechanisms underlying KCs is dictated by their proposed role in sleep maintenance (embodying an arousal with subsequent sleep-guarding counteraction), memory consolidation as well as epilepsy (Colrain, 2005; Halász, 2005; Si et al., 2010). The hereby observed iKCo may reflect subcortically evoked cortical responses rather than cortical spontaneous activity, as is the case for KC. Thus, during the occurrence of KCs, a window of opportunity for subcortical afferents to reach cortex and bias its neurons toward the up state may be offered. The ensuing shift to the latter—concomitant with the positive phase of the KC—may explain the short duration of the iKCo. The iKCo could in turn provide excitatory input to pacemaking circuits in thalamus, thus recruiting other thalamocortical sectors as well (topographical shift of maxima) and after some delay increase its frequency. These effects are transient as in longer lasting iKCos they tend to reverse (iKCo with 3 waves) or even to reverse and repeat themselves (iKCo with 4 waves or with alpha rhythm), suggesting a ceiling, which is different in each subject. If even longer, then the thalamocortical input will continue, resulting in KCs followed by alpha waves and microarousals.

The results also reveal a dynamic relationship between the theta and alpha rhythmic oscillation. Research in animals has so far provided data that support such a relation, as it has been shown that it is an intrinsic property of thalamic neuronal populations to competitively shift between two frequencies of oscillation, the theta, and the alpha (Hughes and Crunelli, 2007; Crunelli and Hughes, 2010). As also in humans (De Gennaro et al., 2001; Niedermeyer, 2004), in cats the cortical alpha rhythm of drowsiness is replaced by theta band oscillations during sleep onset (Hughes et al., 2004). Both the theta and the alpha rhythm in cats have been shown to be highly correlated with high threshold burst firing of individual pools of neurons for each rhythm, when they are sufficiently depolarized (Bouyer et al., 1982; Hughes et al., 2004; Hughes and Crunelli, 2005). These neuronal groups modify their firing frequency between the theta and the alpha frequency bands by acting one upon another with a difference in phase, evidently via gap junctions (Davidson and Baumgarten, 1988; Hughes and Crunelli, 2007). The frequency modulation of the iKCo between the theta and the alpha band may represent thalamic interactions among such neuronal pools during human sleep.

Regarding a putative functional role of iKCo, indirect evidence from evoked KCs has suggested that spontaneous KCs are linked to increases in information processing (Colrain, 2005). Also alpha band activity in sleep correlates to increased sensitivity to environmental noise (McKinney et al., 2011). Is it possible that the described iKCo helps starting information processing of afferent sensory signals? The present study resulted in several important observations that may highlight the physiological role of the iKCo: (1) iKC oscillations follow a pattern of spatial shift of amplitude maxima across the anterio-posterior axis (which was found highly reproducible in the 2- and 3-peak groups), in contrast to the rather systematic and without spatial shift frontal distribution of the slow KC wave, and (2) iKC oscillations exhibit a transient spectral shift pattern between the theta and alpha zone characterizing the instantaneous frequency sequence of each consecutive wave. The second finding is comprised of two phases: (1) an initial phase where the instantaneous frequency of every successive wave shifts from the high-theta band and enters the alpha band, and (2) a late phase where the instantaneous frequency shifts back to the initial spectral limits (either theta or alpha band depending on the iKCo group). The combination of the two main observations potentially support an arousing nature of the phenomenon; an oscillation that tends to transiently adopt higher frequencies (to be transformed from theta to alpha), while at the same time braking away from the global synchrony of the KC (shifting across the anterio-posterior axis of the cortex).

We recently reported that sleep spindles were always interrupted upon KC coincidence and most often resumed as higher frequency spindles afterwards (Kokkinos and Kostopoulos, 2011). The present study complemented the previous report and showed that the oscillation appearing during the KC has labile spatial and spectral features and is independent of the main KC slow wave. The iKCo may be an arousing element, while the KC main negative slow wave and the often following fast spindles are sleep defending elements (Halász et al., 1985; Halász, 2005; Dang-Vu et al., 2010) maintaining sleep as long as the arousing stimuli represented by the iKCo did not pass some threshold of saliency or relevance to the subject. The term “complex” appears to be increasingly justified in terms of the multiple EEG features as well as the underlying mechanisms and hypothesized role of KC.

### Conflict of interest statement

The authors declare that the research was conducted in the absence of any commercial or financial relationships that could be construed as a potential conflict of interest.
